# Matrigel-based organoid culture of malignant mesothelioma reproduces cisplatin sensitivity through CTR1

**DOI:** 10.1186/s12885-023-10966-4

**Published:** 2023-05-31

**Authors:** Fumiya Ito, Katsuhiro Kato, Izumi Yanatori, Yuki Maeda, Toyoaki Murohara, Shinya Toyokuni

**Affiliations:** 1 Department of Pathology and Biological Responses, Nagoya University Graduate School of Medicine, 65 Tsurumai-Cho, Showa-Ku, Nagoya, 466-8550 Japan; 2 Department of Cardiology, Nagoya University Graduate School of Medicine, 65 Tsurumai-Cho, Showa-Ku, Nagoya, 466-8550 Japan; 3 Department of Molecular and Cellular Physiology, Graduate School of Medicine, Kyoto University, Sakyo-Ku, Kyoto, 606-8501 Japan; 4 Center for Low-Temperature Plasma Sciences, Nagoya University, Furo-Cho, Chikusa-Ku, Nagoya, 464-8603 Japan

**Keywords:** Organoid, CTR1, Mesothelioma, EGFR, Asbestos, Carbon nanotube

## Abstract

Organoids are a three-dimensional (3D) culture system that simulate actual organs. Therefore, tumor organoids are expected to predict precise response to chemotherapy in patients. However, to date, few studies have studied the drug responses in organoids of malignant mesothelioma (MM). The poor prognosis of MM emphasizes the importance of establishing a protocol for generating MM-organoid for research and clinical use. Here, we established murine MM organoids from *p53*^+/-^ *or wild-type C57BL/6 strain* by intraperitoneal injection either with crocidolite or carbon nanotube. Established MM-organoids proliferated in Matrigel as spheroids. Subcutaneous injection assays revealed that the MM-organoids mimicked actual tissue architecture and maintained the original histological features of the primary MM. RNA sequencing and pathway analyses revealed that the significant expressional differences between the 2D- and 3D-culture systems were observed in receptor tyrosine kinases, including IGF1R and EGFR, glycosylation and cholesterol/steroid metabolism. MM-organoids exhibited a more sensitive response to cisplatin through stable plasma membrane localization of a major cisplatin transporter, copper transporter 1/Slc31A1 (Ctr1) in comparison to 2D-cultures, presumably through glycosylation and lipidation. The Matrigel culture system facilitated the localization of CTR1 on the plasma membrane, which simulated the original MMs and the subcutaneous xenografts. These results suggest that the newly developed protocol for MM-organoids is useful to study strategies to overcome chemotherapy resistance to cisplatin.

**Supplementary Information:**

The online version contains supplementary material available at 10.1186/s12885-023-10966-4.

## Introduction

Organoid technology bridges the gap between traditional two-dimensional (2D) cell line cultures and in vivo models [1–3]. Organoids are small 3D tissues that can be generated from embryonic stem cells (ESCs), induced pluripotent stem cells (*i*PSCs), adult stem cells and cancer cells [4]. Organoids maintain intrinsic properties of the primary tumors, including self-renewal, multilineage differentiation, signaling nodes and histological structures. In addition to displaying a specific morphology, such as microvilli and tight junction, organoids exhibit apical-basal cellular polarity. Regarding cancer biology, organoids are expected to present various applications in translational studies under precision medicine.

Malignant mesothelioma (MM) is an extremely lethal tumor [5], arising from the mesothelial cells of pleura, peritoneum or pericardium, which has been linked to asbestos exposure [6, 7]. Recently, we identified the iron-rich mutagenic milieu, resulting from the accumulation of asbestos-fed macrophages with myofibroblasts, leading to granuloma [8, 9]. This granuloma formation is accompanied by increased stiffness and extracellular matrix deposition [10], and the stiffness indeed contributes to the malignant phenotype in tumor biology [11, 12]. Therefore, experimental cultures may expect a similar stiffness for accurate results [13]. One of the major problems in cancer treatment is the presence of cancer stem cells (CSCs). CSCs are resistant to chemotherapy and are involved in cancer initiation and metastasis [14, 15]. Therefore, many studies have focused on the biology of CSCs to target them in cancer treatment. Although poor clinical prognosis of MM requires novel therapeutic approaches, not much is thus far known on the characteristics of both normal mesothelial and MM stem cells.

Cisplatin has been widely used in cancer therapy, including the first line chemotherapy for MM [5]. Homozygous deletion of the 9p21 locus in humans that harbors genes, encoding *CDKN2A and CDKN2B,* are the most common genetic alterations in MM [7]. Of note, *miR-31* is located also at the same locus, which promotes cisplatin sensitivity through simultaneous loss of *miR-31* [16]. CTR1 (SLC31A1) contributes to cisplatin uptake in the renal tubular cells during nephrotoxicity, a major side effect of cisplatin [17]. Mammalian *CTR1* mRNA expression has been observed in all the examined tissues with kidney and liver at high levels and with brain and spleen at low levels. Reportedly, cisplatin is preferentially taken up through the basolateral membrane in the renal tubular cells [18–20]. Cisplatin uptake is thus associated with cellular polarity. Accordingly, a model MM system, replicating cellular polarity, is essential to further understand the mechanisms of cisplatin resistance in MM. However, there are thus far no established protocols for organoid development in MM.

Here we started from establishing a protocol to generate MM organoids from mice. Mouse MM organoids is effective as pre-clinical models because MM is a rare tumor in Japan with the incidence of ~ 2,000 per year in 126 million population [7]. Fortunately, human and mouse CTR1 are highly homologous. After the first 37 residues, only four residues in mouse Ctr1 (mCtr1) are different from those of human CTR1 (hCTR1) [21]. These newly generated MM-organoids would potentially provide us with an understanding of cisplatin-transport dynamics in MM, thereby highlighting the merits of organoid culture to study clinical responses to cisplatin treatment and the chemotherapy resistance.

## Materials and methods

### Materials

The materials and kits used are as follows. EMEM (Wako, 051–07,615, Osaka, Japan), FBS (Biowest, S1810-500; Nuaillé, France), Tet system Approved FBS (TaKaRa, 631,107, Shiga, Japan), Autofluorescence Quenching Kit (Vector, SP-8400, Burlingame, CA), AurumTM Total RNA Mini Kit (BioRad, 64,337,836, Hercules, CA), NEBNext Ultra II Directional RNA Library prep kit for Illumina (BioLabs, E7760S, Boston, MA), NEBNext Multiplex Oligos for Illumina (BioLabs, E6440S), anti-CTR1 (CST, #13,086, Danvers, MA, Novus, #NBP2-36,573), anti-Flag (MBL, PM020, M185-3L, Nagoya, Japan), anti-golgin97 (CST, #13,192), anti-mouse mesothelin (IBL, #28,127, Gunma, Japan), anti-WT1 (Thermo Fischer Scientific, MA5-32,215), anti-ITGβ1 (CST, #9699), anti-IGFR (Proteintech, 20,254–1-AP, Chicago, IL), anti-β-catenin (CST, #8480 T), anti-GFP (MBL life science, #598), anti-mouse IgG Alexa488 (ThermoFischer Scientific, A-11108), anti-rabbit IgG Alexa 568 (Thermo Fischer Scientific, A-11011), anti-rabbit IgG Alexa 633 (Thermo Fischer Scientific, A-21070) CF®568 Phalloidin (biotinum,00064 T), Receptor Tyrosine Kinase Antibody Sampler Kit (CST, #42,344), Epithelial-Mesenchymal Transition (EMT) Antibody Sampler Kit (CST, #9782), anti-β-actin (Sigma Aldrich, P2120, St. Louis, MO) Advanced F12/DMEM (Thermo Fischer Scientific, 12,634,010), recombinant Murine R-Spondin-1 (PeproTech Inc., 315–32, Cranbury, NJ), recombinant Murine Noggin (PeproTech Inc., 250–38), recombinant Murine EGF (PeproTech Inc., 315–09), recombinant Wnt3A (PeproTech Inc., 315–20-2ug), recombinant FGF-basic (PeproTech Inc., 450–33), B-27™ Supplement (50x) (Thermo Fischer Scientific, 17,504,044), N-2 Supplement (100x) (ThermoFischer Scientific, 17,502,048), A8301 (Peprotech Inc., 9,094,360), N-Acetyl-L-cysteine (Wako, 017–05,131) CTS™ GlutaMAX™-I Supplement (ThermoFischer Scientific, A1286001), CultureSure® Y-27632 (Wako, 030–24,021) and Corning® Matrigel® Growth Factor Reduced (GFR) (Corning, 354,230) were used.

### Cell experiments

Mouse mesothelial cell lines were established from the *C57BL/6* mice, using the previously described enzymatic method [22–24]. Briefly, omentum and major intraperitoneal organs, including stomach, spleen and pancreas, were excised from 6 ~ 8-week-old mice, which was followed by careful separation of mesothelium, using scissors. Approximately 20 to 30 mesothelial fragments were isolated from each isolated serosal preparation, of which roughly 80% were found to attach to the Nunclon™ Delta-treated culture plate and to start proliferation with DMEM containing 10% FBS after overnight culture. The mesothelial cells were immortalized using SV40 as well as small and large T antigen (Addgene#22,298). Mouse MM cell lines were established also from *p53*-heterozygous knockout (*p53*^+/-^ )mice as described below. All the cell lines were cultured under standard culturing conditions (37 °C, humidified atmosphere, 5% CO_2_) in EMEM containing 10% FBS and 1% antibiotic–antimycotic (Gibco). Cells were frozen in CellBanker (TakaraBio), thawed and used for experiments within two months.

### MM carcinogenesis model in mice

MM carcinogenesis in mice were performed as previously described with some modifications [25–27]. *P53*-heterozygous knockout (*p53*^+/-^ mice were obtained from RIKEN BRC (RBRC01361 [28]) and were maintained by mating with normal *wild-type C57BL/6* female mice (SLC, Shizuoka, Japan). Fourteen *p53*^+/-^ and 10 *p53*^+/+^ male mice at the age of 6 ~ 8 weeks were injected intraperitoneally (*ip*) with a single dose either of 3.0 mg crocidolite (UICC) or 1.5 mg of multi-walled carbon nanotube of 50 nm-diameter (NT-50; VGCF-s) [29] in 3 ml of 0.5% bovine serum albumin/saline suspension. The vehicle solution (3 ml) was injected into 9 mice as negative controls. The mice were euthanized by cervical dislocation under isoflurane-anesthesia when they revealed massive ascites or 5% weigh loss in a week. Mouse that did not exhibit these phenotypes were euthanized after approximately 1y (365 d) had passed from the initial injection of fibers. All the animal experiments were approved by the animal experimental and ethics committee of Nagoya University Graduate School of Medicine (M220346-004, 2022/03/18).

### Isolation of MM cells

Immediately after euthanizing the animals, tissue fragments of ~ 100–300 mm^3^ were excised from the induced MM. Non-necrotic areas with solid growth were selectively collected and processed as follows. Tissue fragments were cut into 2–3 mm pieces, washed with cold PBS several times and finally dissociated into small cell clusters or single cells through digestion with 2 U/ml dispase II and 1 mg/ml collagenase P (SigmaAldrich, 11,213,857,001) for 30 min at 37 °C. Dissociated cells were washed again with PBS and recovered as a pellet by centrifugation (500 × *g* for 5 min). If necessary, further digestion was performed using Accumax (Innovative Cell Technologies, San Diego, CA) for 5 min at 37 °C, washed with ice-cold PBS and recovered as a pellet by centrifugation (500 × *g* for 5 min). Erythrocytes were removed using hypotonic buffer (168 mM NH_4_Cl, 10 mM KHCO_3_, 81.8 µM EDTA-4Na). The number of wells to seed the MM cells was determined depending on the number of cell pellets recovered. Typically, 2 to 4 wells in a 48-well plate were used to initiate the primary organoid culture.

### Organoid culture of MM cells

**Fig. 1:**
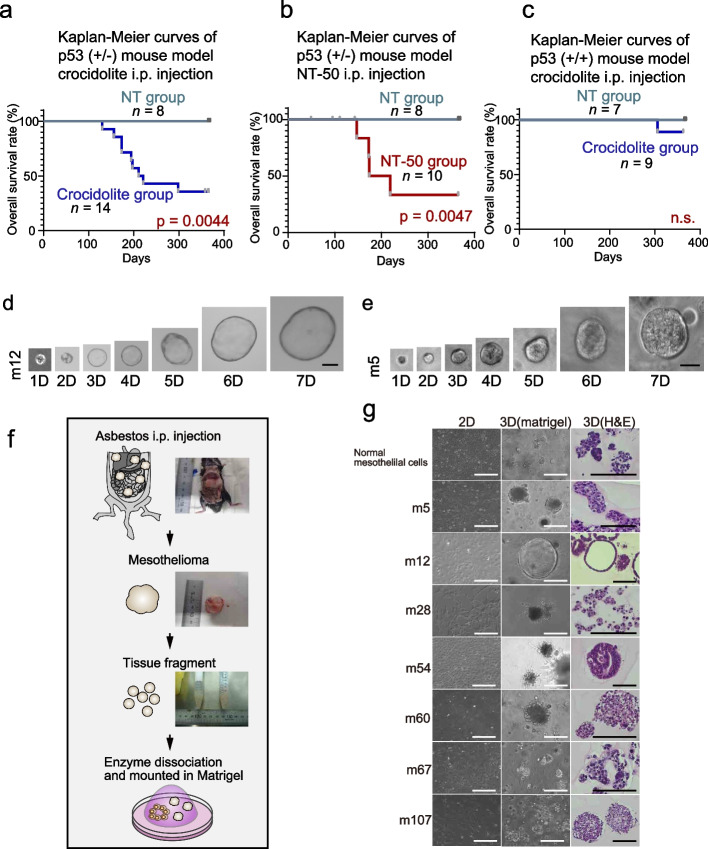
Preparation of malignant mesothelioma (MM) organoids. **a**–**c** Kaplan Meier analysis for the MM mouse models, describing the overall survival probability in p53( +/-); blue line, crocidolite group; red line, NT-50 group; gray blue lines, the untreated control groups. **d**, **e** Time course culture of the epithelioid (m12) and sarcomatoid (m5) MM organoids in 1–7 days (scale bar = 50 μm). **f** Schematic illustration of the protocol to establish MM organoids from the dissection of mouse MM to matrigel-based 3D culture. **g** Phase-contrast and H&E-stained images of normal mesothelial cell or MM-2D and 3D cultures (scale bar = 100 μm)

The culture media used for organoids was advanced DMEM/F12 (Thermo Fischer Scientific) supplemented with 50 ng/ml recombinant murine EGF (Peprotech), 100 ng/ml recombinant murine R-spondin1 (R&D, Minneapolis, MN) and 100 ng/ml recombinant murine Noggin (Peprotech), 10 µM Y-27632 (Wako). All the following experiments used this organoid medium. Organoids were cultured according to the general organoid-culture method established previously (Fig. 1f) [30]. The cells were resuspended in 250 µl of media and plated on 25 µl of solidified Matrigel (BD Bioscience, Franklin Lakes, NJ) per well in a 48-well plate and incubated overnight at 37 °C. Subsequently, floating non-viable cells were discarded along with the media. In instances when floating tissue fragments and cell aggregates appeared to contain numerous viable cells, they were recollected and digested with Accumax (Innovative Cell Technologies, San Diego, CA) for 5 min at 37 °C along with vigorous pipetting. For each passage, the cells were cultured until 70–80% confluency, from which 5 × 10^5^ ~ 2 × 10^6^ cells were typically harvested. Depending on the proliferation rate, the cells were diluted at 1:1 to 1:4 with culture media. For each passage, organoids, Matrigel and media were collected directly using a cell scraper, washed with PBS and then dissociated into single cells using Accumax treatment for 5 min at 37 °C along with vigorous pipetting. MM-derived organoids were cryopreserved in Bambanker (GC Lymphotec, Tokyo, Japan) supplemented with 10 µM Y-27632 and stored at − 80 °C until use. The organoids were thawed and used for experiments within two months (passenger times < 20).

### Pathological analysis

Organoids were first isolated by de-polymerizing the Matrigel with Cell recovery Solution (BD Bioscience, Franklin Lakes, NJ) and then resuspended in iPGell (Genostaff, Tokyo, Japan). The iPGell-embedded organoids or the resected tumors were fixed in 4% paraformaldehyde (PFA), dehydrated and then embedded in paraffin. The formalin-fixed paraffin-embedded (FFPE) samples were cut into sections with 4-µm thickness and then stained with hematoxylin and eosin (H&E), alcian blue (pH 2.5) or mucicarmine staining. Hyaluronic acid was detected by hyaluronic acid binding protein using the manufacturer's protocol (Amsbio, AMS.HKD-BC41, Abingdon, UK). Immunohistochemical stainings were performed with a Leica BOND Max (Leica Microsystems, Wetzlar, Germany) as described [31].

### Immunofluorescence staining

Cells were plated on coverslips with or without matrigel. After 18–24 h incubation, cells were fixed with 4% PFA for 10 min/4℃, permeabilized for 5 min in 0.2%Triton X-100 and then blocked with 3% BSA in PBS for 15 min. Cells were stained with specific antibodies namely anti-FLAG, anti-ITGB1, and anti-CTR1 at a dilution following the manufacture`s recommendation for 30 min at room temperature. Then, secondary antibodies conjugated to Alexa 488, Alexa 568, or Alexa 647 were used at 1:1000 for 30 min at room temperature with Hoechst 33,342 at 1:10,000. Following a final wash in H_2_O, glass coverslips were mounted with VECTASHIELD Antifade Mounting Medium (H-1900) (Vector Lab Inc., Newark, CA). Images were obtained using Olympus SpinSR10 confocal microscope and 100x/1.4NA oil immersion objective or IX81 inverted microscope (Olympus) and 60 × /1.42 NA oil objective lens. Images were analyzed using the image processing package, Fiji (https://fiji.sc/).

### Cloning of mCTR1

The amino acid coding regions of mouse CTR1, was amplified using cDNA from mouse normal mesothelial cells. mCTR1 was PCR-cloned into pCIG3 modified vector as the same of vector construction section. Additionally, mCTR1 was PCR-amplified to insert a FLAG epitope (NH_2_-DYKDDDDK-COOH) at the amino terminus of mCTR1. Mutagenic primers were designed to introduce single amino acid substitution at amino acids N23D and N23Q (Table 1). Mesothelial cells, MM cells and MEF cells with *CTR1* overexpression or mutated *CTR1* were established, using either a pCIG3 modified lentiviral vector (Addgene, #78,264) or pcDNA3.2.

**Table 1:** Primer sequences for cloning and mutagenesis of mCTR1

Primer	Primer orientation	Nucleotide sequence
N-terminal Flag mCTR1	Forward	5'-GAATTCGCCACCATGGACTACAAAGACGATGACGACAAGAACCA-3'
	Reverse	5'-GGATCCTCAATGGCAGTGCTCTGTGA-3'
mCTR1	Forward	5'-GAATTCGCCACCATGAACCATATGGGGATGAACCATATGG-3'
	Reverse	5'-GGATCCTCAATGGCAGTGCTCTGTGA-3'
N23D	Forward	5'-ATTACCATGCCACCTCACCACCAC-3'
	Reverse	5'-GTCGTCGTCCGTGTGGTTCATACCCATATG-3'
N23Q	Forward	5'-ATTACCATGCCACCTCACCACCAC-3'
	Reverse	5'-CTGGTCGTCCGTGTGGTTCATACCCATATG-3'

Primers were designed for generating different mouse CTR1 constructs, which were ligated into the appropriate epitope-tag sequences and then cloned into pcDNA3.2 vector

### Cell proliferation and drug sensitivity assays

Regarding in vitro assays, organoids were digested in Accumax for 5 min at 37 ℃ and thereafter in cell recovery solution for 20 min on ice. The dissociated cells were counted using the Countess II FL (Thermo Fischer Scientific). For the cell proliferation assay, 2 × 10^4^ cells were plated in duplicate in a 24-well plate containing solidified Matrigel to form organoids. Data were collected at day 7 and normalized to the corresponding value of day 1. Regarding the drug sensitivity assay, 5 × 10^3^ single cells were plated into a 96-well plate in triplicate. At 48 h after plating, cisplatin (Wako) was added at five serially diluted concentrations, varying from 5 to 100 nM or from 1 to 100 µM. Cell viability was analyzed using the AlamarBlue® Cell Viability Reagent (Thermo Fischer Scientific) after 24 h-incubation.

### Tumorigenicity assay

Immunodeficient nude mice *KSN/Slc*^*nu/nu*^ were purchased from SLC Japan (4 ~ 6 weeks old; Shizuoka, Japan). The tumorigenicity assay was conducted as described previously [30]. Briefly, tumor-derived organoids were collected at 70–80% confluency, and an aliquot of 1/10 the volume was completely dissociated using Accumax for cell counting with the Countess FL Automated Cell Counter. For each organoid, 1 × 10^6^ cells were resuspended in 100 µl of advanced DMEM/F12, mixed with 100 µl of Matrigel at 1:1 ratio and thereafter injected subcutaneously (*sc*) into the dorsal skin of the nude mice. Tumor development was monitored for 4 weeks.

### RNA sequencing and data analysis

2D-culture cells were cultured in appropriate media and harvested by trypsinization. 3D-culture cells were cultured in organoid culture conditions described above. Total RNA was isolated using the AurumTM Total RNA Mini Kit (BioRad) according to the manufacturer’s protocol. RNA quality was assessed using a 2100 BioAnalyzer (Agilent). In total, 100 ng of RNA was used to prepare sequencing libraries, using NEBNext Ultra II Directional RNA Library Prep with Sample Purification Beads (NEB), according to the manufacturer’s instructions. Libraries were validated using the BioAnalyzer and quantified by qPCR and Qubit Fluorometric Quantitation (Thermo Fischer). Novaseq 6000 (Illumina), which generates 150-bp pair-end reads, was used. Data analysis of RNA-seq was performed as previously described with some modifications [9, 32]. Quality of raw sequence data was assessed using FastQC (Version: FastQC 0.10.0). FASTQ files were trimmed for adaptors and Phred score (> 20) using TrimGalore! (0.6.4). Trimmed sequenced reads were aligned to the mouse genome assembly (GRCm39 Ensembl release 103) using STAR (2.7.9a). Aligned reads were used to quantify mRNA with HTSeq-count (version 0.12.4). Differential gene expression analysis of paired-comparison between 2D-culture and 3D-culture was performed using edgeR package on protein-coding genes. Genes were considered as differentially expressed when the FDR-adjusted *P* value was below 0.05. Gene ontology analysis was performed using the online bioinformatic tool, Database for Annotation, Visualization and Integrated Discovery (DAVID, v6.8) and Enrichr (https://maayanlab.cloud/Enrichr/). Heat maps were generated using either R package pheatmap or heatmap3.

### Immunoblot analysis

Protein extraction and sodium dodecyl sulfate–polyacrylamide gel electrophoresis (SDS-PAGE) were performed according to the previously established protocol [33]. Cell line samples were lysed using the RIPA buffer (150 mM NaCl, 50 mM Tris–HCl (pH 7.4), 1% Nonidet P-40, 1% SDS, 0.5% deoxycholate), containing PierceTM Protease and Phosphatase inhibitor (Thermo Scientific). Subsequently, they were homogenized by super-sonication (UD-100, TOMY, Tokyo, Japan) and clarified by centrifugation at 18,000 × g for 30 min at 4 °C. The total protein concentration of the lysates was quantified using the Protein Assay Bicinchoninate kit (Nacalai tesque, Kyoto, Japan).

### Statistical analyses

Statistical analyses were performed using the Graphpad Prism software (version 8.4.3) and the R statistical environment (http://r-project.org). All data are presented as means ± SEM, unless indicated otherwise. Data were tested for normal distribution with unpaired two-tailed *Student*’s *t*-test (if two samples have equal variances) or *Welch*'s *t*-test (if two samples have unequal variances) to determine statistical significance between the two groups. Statistical significance among more than two groups were analyzed using one-way *ANOVA* along with *Tukey*'s multiple comparison test to assess statistical significance with a 95% confidence interval. P < 0.05 was considered statistically significant, unless otherwise specified. All the experiments were independently repeated at least three times, unless otherwise mentioned.

## Results

### Establishment of organoids from mouse MM

MM organoids were generated using mouse MM models by intraperitoneally injecting mice with either asbestos (crocidolite) [25–27] or carbon nanotubes (NT-50) [29] as described previously. MM incidence was 42.9% in crocidolite-treated *p53*^*+/-*^ mice (Table 2), which was similar to the result of a previous report [34]. MM was assessed to be the main cause of death in the crocidolite-treated mice whereas ileus caused severe peritonitis with adhesion, primarily leading to death, in the NT-50-treated mice (Fig. 1a–c). Attempts to establish MM organoids from the MM tissue or ascites resulted in an establishment and successful propagation of organoids in 7 of the 11 MM cases (Table 2 and Fig. 1f, g). To test whether the organoids have a self-renewal potential, we performed single-cell seeding assay, where single-cell suspensions could generate MM organoids (Fig. 1d, e). The MM organoids revealed two distinct patterns. Most organoids formed a spherical pattern whereas only one MM-organoid (m12) formed a budding pattern (Fig. 1d–g). MM-organoids derived from MM tissue were confirmed to propagate at least for 6 months, tolerating freeze and thaw (data not shown). These results indicated that mouse MM-derived organoids can function as a useful resource for research on MM (Table 3).

**Table 2:** MM incidence and organoid lines derived from *p53*^+/-^ and *wild-type C57BL6* mouse

Mouse genotype	Material	Total number	Mesothelioma incidence		Established organoids	
			**Number**	**%**	**Number**	**%**
*P53 +/-*	Crocidolite	14	6	42.9	5	83.3
	NT50	10(3)	3	42.9	1	33.3
	NT	8	0	0.0	0	ND
*P53 + / +*	Crocidolite	10(1)	1	11.1	1	100.0
	NT50	4(2)	1	50.0	0	0
	NT	7	0	0.0	0	ND

The total number includes mice died from ileus resulting from peritonitis in parentheses. Mesothelioma incidence: mesothelioma/[total number-ileus] (%). Established organoid: the number of established organoids/number or mesothelioma incidence (%). *NT* Carbon nanotube, *NT* No treatment, *ND* Not determined. Refer to text for details

**Table 3:** Histological summary of mice with exposed materials and the days of tumor development

Organoid ID	Material	mouse genotype	Mesothelioma incidence	Histology		
			**Days**	**Subtype**	**MSLN**	**WT1**
m5	Crocidolite	*P53 +/-*	211	Biphasic	+	+
m12	Crocidolite	*P53 +/-*	156	Biphasic	-	-
m28	Crocidolite	*P53 +/-*	221	Sarcomatoid	+	+
m54	Crocidolite	*P53 +/-*	173	Sarcomatoid	+	-
m60	Crocidolite	*P53 +/-*	194	Biphasic	-	-
m67	Crocidolite	*P53 + / +*	139	ND(Ascites)	ND(Ascites)	ND(Ascites)
m107	NT50	*P53 +/-*	174	Sarcomatoid	+	+

*NT* Carbon nanotube, *ND* Not determined. Refer to text for details

### Epithelial growth factors regulate MM stemness and proliferation in organoids

**Fig. 2:**
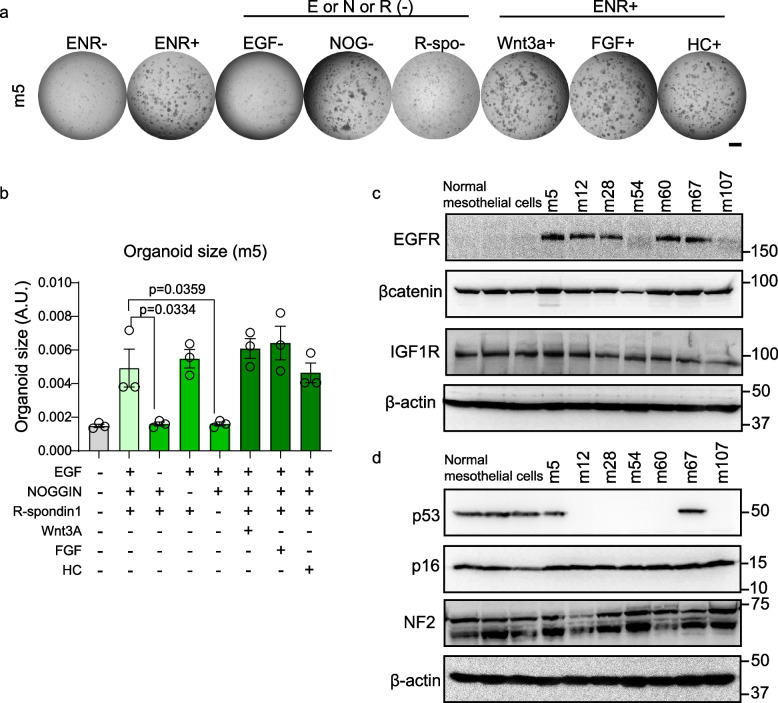
MM organoids require epidermal growth factor through EGFR. **a** The images of MM organoids at 7 days (scale bar = 1 mm). MM-organoids were incubated with or without growth factors. Organoid growth factors: EGF (E), NOGGIN (N or NOG), R-Spondin1 (R or R-spo), Fibroblast Growth Factor (FGF), Hydrocortisone (HC). **b** The sizes of individual MM-organoids after co-incubation with EGF, NOGGIN, R-spondin1, Wnt3A, Fibroblast Growth Factor (FGF) and/or Hydrocortisone (HC). Gray, no growth factor; light green, all essential growth factor (ENR; EGF, NOGGIN and R-spondin1); green, ENR minus single essential growth factor; dark green; ENR plus other growth factor. **c** Immunoblot analysis of receptor tyrosine kinase in the 2D normal mesothelial and MM cell lines. **d** Immunoblot analysis of tumor suppressor proteins in the 2D normal mesothelial and MM cell-lines

In order to test the dependence of MM-derived mesothelial organoids on the crucial growth factors supplemented in the organoid growth medium, organoids were dissociated to single cells and resuspended in organoid media either with or without the crucial growth factors. EGF, Noggin and R-spondin1 are widely accepted as most essential factors in the organoid medium. We also tested additional factors such as Wnt3a, FGF and hydrocortisone (HC) [35–38]. Compared to the m5 organoid line (Table 3), the m12 organoid line grew faster, independent of all the growth factors except epidermal growth factor (EGF) (Fig. 2a, b, S2a, b). In contrast, the m5 organoid line required both EGF and R-spondin1 (Fig. 2a, b). EGF-receptor (EGFR) is overexpressed in various epithelial malignancies and in pleural MM [39, 40]. In order to study the differences between these newly established MM cell lines and normal mesothelial cells, we performed immunoblotting analysis on the MM-associated oncoproteins and the tumor suppressor proteins [41]. Expression of β-catenin and insulin growth factor receptor 1 (IGFR) was not altered in mouse MM cell lines. However, EGFR was expressed in some MM cell lines (Fig. 2c). Furthermore, p53 tumor suppressor protein was at the undetectable level in five MM cell lines (Fig. 2d). These results suggest that most of the newly derived MM cells depends on EGFR pathway activation and/or loss of p53 and MM cells required the EGF to grow as organoid.

### Implants of MM-organoids maintain characteristics of primary MM

**Fig. 3:**
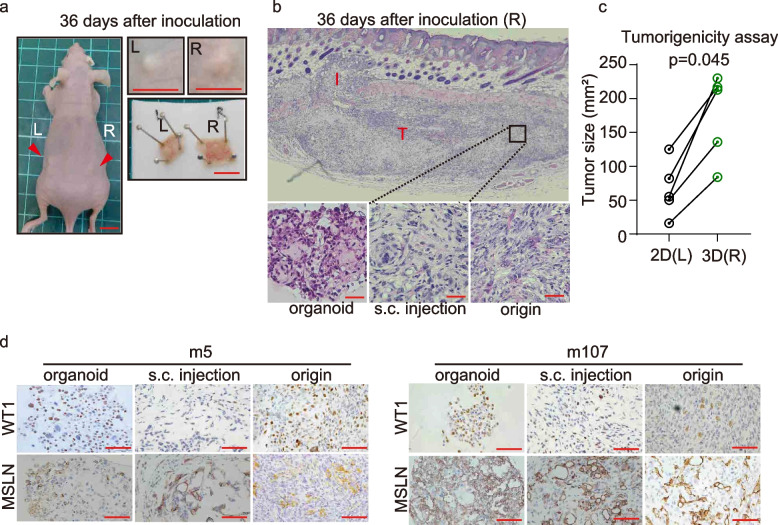
Subcutaneous (*sc*) injection of MM-organoids and 2D MM cell lines. **a** *SC* inoculation to nude mice with 2D MM cell lines (m107), left (L), and MM-organoids, right (R), respectively. Mice were euthanized at day 36  after inoculation. Left image, whole-body image from the back. The right upper image, lateral view of the tumor. The right lower image, intradermal view of the tumor. Arrowheads and red lines, tumor (scale bar = 10 mm). **b** Histological images of the H&E-stained right tumor nodule by m107 cell line (scale bar 20 μm). I, invasion; T, tumor nodule. **c** Left (2D MM cell lines) and Right (MM-organoid) tumor size (mm.^2^). **d** Histological presentations with immunohistochemical staining of mesothelial markers (WT-1, MSLN). Left images, m5 mice origin. Right images, m107 mice origin (scale bar 100 μm)

We then evaluated the capacities of mouse MM organoids to retain mesothelial characteristics. Secretion of hyaluronic acid (HA) is one of the characteristics of MM [42]. The retention of HA secretion by MM organoid was confirmed by detection with acidic mucin staining or HA binding protein in a similar fashion between the mouse MM-derived organoids and MM tissues (Fig. S3). In order to gain further insight into the MM organoid culturing system, the MM-organoids and 2D MM cell lines were simultaneously injected into the bilateral dorsal skin of immunodeficient mice (Fig. 3a). We used m5 and m107 cell line because these two lines are typical mouse MM by the expression of WT1 and mesothelin (MSLN) (Table 3) and EGFR or P53 expression status were completely opposite in the two cell lines (Fig. 2c, d). At 36 days after the *sc* injection, histology of both the implants revealed a similar solid growth pattern (Fig. 3b). However, proliferation of the MM organoids was significantly faster than that of the 2D MM lines (Fig. 3a, c). Immunohistochemical analyses of the implants revealed the expression of WT1 and MSLN, confirming the maintenance of the original characteristics (Fig. 3d). These results suggest that MM organoids can capture the in vivo characteristics of MM developed by crocidolite or CNT-50 injection in mice.

### RNA sequencing analysis revealed expressional alteration between MM organoids and 2D-culture

**Fig. 4:**
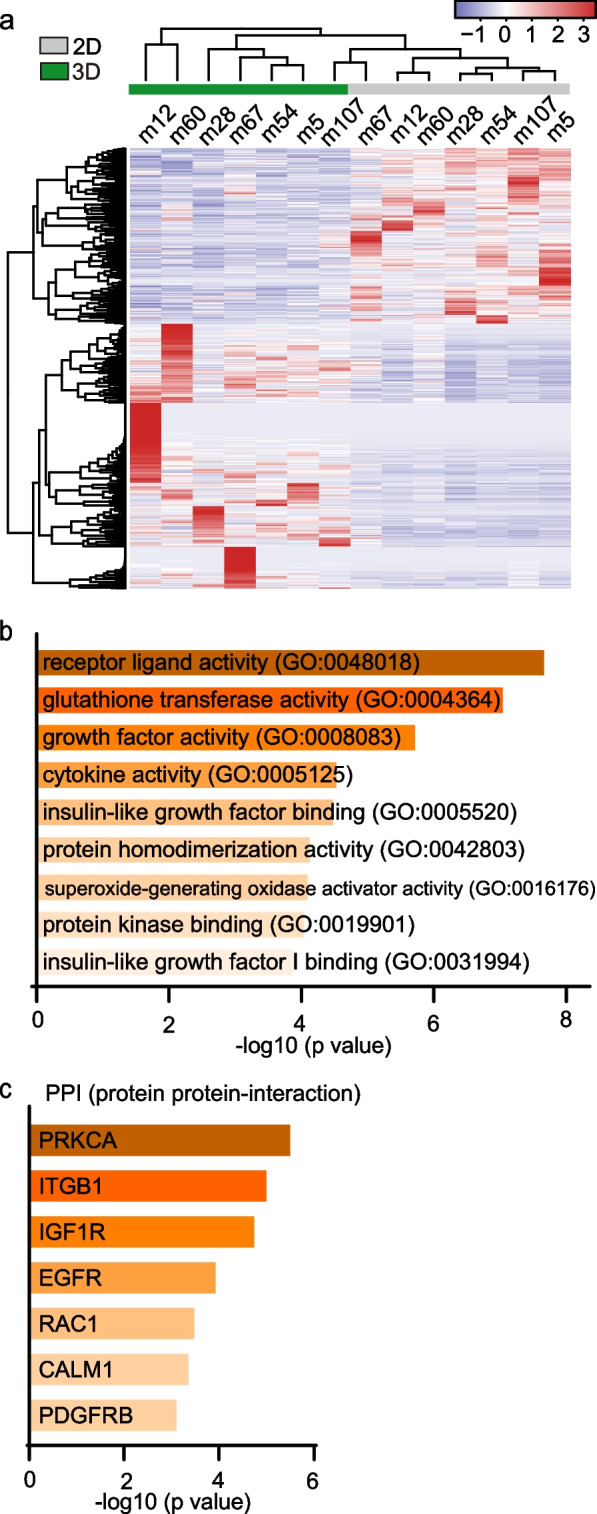
Transcriptome profiling of malignant mesothelioma (MM)-derived organoids and 2D-culture. **a** Comparison of transcriptional profiles of mesothelioma cells obtained from traditional 2D culture (2D, gray bar) and Matrigel-based 3D culture (3D, green bar). Red and blue colors indicate upregulation and downregulation, respectively. **b**, **c** Top gene ontology (GO) using Enrichr molecular function and PPI Hub proteins terms related to differentially expressed genes between 2D and 3D cultures of mouse MMs

We further investigated the differences between MM organoids and 2D MM cell lines with RNA sequencing analysis. MM organoids and 2D MM cell lines revealed genes in distinct clusters (Fig. 4a). Gene Ontology analysis for biological processes and cellular components linking the gene expressional changes between MM organoids (3D) cells and 2D culture-associated genes indicated that molecular function and Protein Protein-Interaction (PPI) Hub Proteins were associated with the receptor tyrosine kinases, such as IGFR and EGFR (Fig. 4b, c). These results suggest that the different culture systems strongly alter gene expression in MM.

### Sensitivity of MM organoids to cisplatin depends on CTR1

**Fig. 5:**
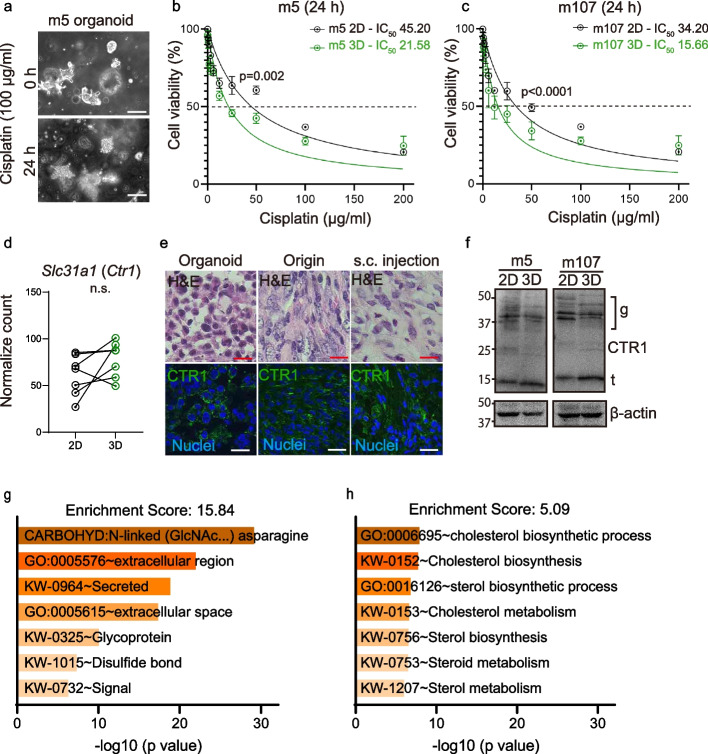
Matrigel mediates high sensitivity to cisplatin. **a** Phase-contrast images of m5 MM-organoids before and after cisplatin (100 μg /ml, 24 h) treatment. The majority of MM organoids die within 24 h after cisplatin treatment. **b**, **c** Cell viability of m5/m107 MM-organoids and 2D MM cell lines in different cisplatin dosage (0.5–200 μg/ml, 24 h). IC50 values (concentration responsible for 50% cell growth inhibition) are shown in each line. Green lines, MM-organoid; black lines, 2D MM-cell lines. **d** *Slc31a1* (*Ctr1*) expression level in MM-organoids and 2D MM cell lines from RNA-sequence data. **e** H&E staining (upper) and immunofluorescent images from m5 MM primary tissue sample, *SC* injection and organoids. CTR1 (Alexa488, green); nuclear staining (Hoechst33342, blue; scale bar = 20 μm). **f** Immunoblot analysis of m5/m107 MM-organoids (3D) and 2D MM cell lines (2D); g, glycosylation (30–50 kDa); CTR1 (25 kDa); t, truncate (15 kDa). **g**, **h** Enrichment analysis using DAVID indicates significant activation of protein glycosylation and lipidation pathways between mesothelioma 2D- and 3D-cultures

In order to assess the usefulness of MM organoids as a pre-clinical model to evaluate the sensitivity to chemotherapeutic agents, we performed cell viability assays after cisplatin treatment (Fig. 5a-c). MM organoids were significantly more sensitive to cisplatin treatment than the corresponding 2D MM cell lines (Fig. 5b, c, S2c, d). A SLC31 family copper transporter, CTR1, is a major cisplatin transporter for uptake and its suppression contributes to chemoresistance [17]. However, *Slc31a1 (Ctr1)* mRNA expression was not significantly different between MM organoids and 2D MM cell lines based on RNA-sequence analysis (Fig. 5d) and the cellular localization of CTR1 was not significantly different as well (Fig. 5e). We found, however, a significant difference in the band number and band-shift on the immunoblot of CTR1 between MM organoids and MM 2D cultures, suggesting difference in glycosylation or possibly lipidation, which revealed a similar pattern between m5 and m107 (Fig. 5f). Our pathway analysis showed that protein glycosylation was strongly affected between MM organoids and 2D MM cell lines (Fig. 5g, h). These results suggest that posttranslational modification of CTR1 protein but not expressional level regulates cisplatin sensitivity.

### Glycosylation and extracellular stiffness control the CTR1 localization

**Fig. 6:**
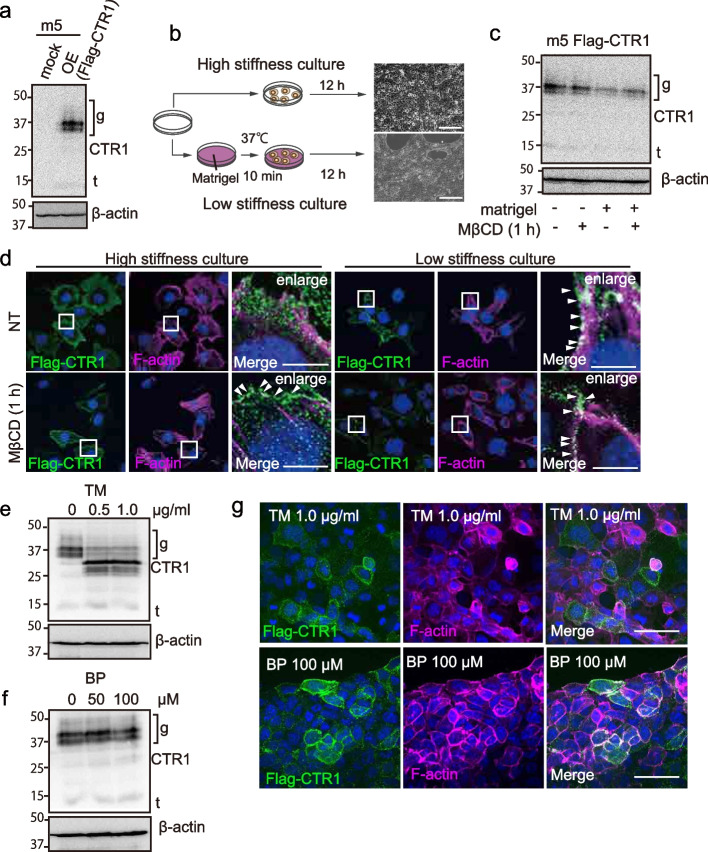
CTR1 localization regulated by extracellular stiffness and glycosylation. **a** Immunoblot analysis of CTR1 in m5-2D MM cell line, comparing mock and overexpression (OE) of flag-CTR1 group; g, glycosylation (30–50 kDa); CTR1 (25 kDa); t: truncate (15 kDa). **b** Schematic images of the culture system. High stiffness culture corresponds to direct plating whereas low stiffness culture corresponds to plating on the Matrigel layer (scale bar = 100 μm). **c** Immunoblot analysis of CTR1. Matrigel, Matrigel layer in the legend (b). MβCD (10 μM, 1 h); g, glycosylation (30–50 kDa); CTR1 (25 kDa); t: truncate (15 kDa). **d** Immunofluorescent images from m5 Flag-CTR1 stably expressed cell line. Left images; cells were cultured on glass slip as high stiffness culture. Right images; cells were cultured on Matrigel layer as a low stiffness culture. Flag-CTR1 (Alexa 488, green), F-actin (CF 568, magenta) and nuclear staining (Hoechst33342, blue; scale bar, 5 μm). **e**, **f** Immunoblot analysis of m5 Flag-CTR1 stably expressed cell after treatment of TM (tunicamycin) or BP (2-bromopalmitate) for 9 h. g, glycosylation (30–50 kDa); CTR1 (25 kDa); t, truncate (15 kDa). **g** Immunofluorescent images from m5 Flag-CTR1 stably expressed cells with after treatment of TM or BP for 9 h. Flag-CTR1 (Alexa 488, green), F-actin (CF 568, magenta) and nuclear staining (Hoechst33342, blue; scale bar: 20 μm). Refer to text for details

One major differences in organoid and 2D-culture is stiffness, which may affect cell metabolism [43]. Protein glycosylation and lipidation regulate its cellular localization [44, 45]. We thus studied the CTR1 localization between MM-organoid and 2D cultures. For this purpose, we established the murine MM cells, stably expressing flag-mCTR1 (Fig. 6a) and used the two different culture systems, high-stiffness culture with plastic bottom and low stiffness culture with Matrigel layer (Fig. 6b). MβCD, a known endocytosis inhibitor, eventually induces membrane localization of CTR1 [46]. After 24 h incubation, CTR1 expression decreased in the low stiffness culture but the MβCD induced no alteration (Fig. 6c). Actin filament (F-actin) is known as a protein localizing to plasma membrane, thus we used F-actin as a marker of plasma membrane (Fig. 6d). Surprisingly, CTR1 localized in the cytosol with 2D MM cell lines whereas 2D MM cell lines plated on Matrigel exhibited CTR1 localization on the plasma membrane in a similar fashion to MβCD application (Fig. 6d), suggesting that extracellular stiffness regulates CTR1 localization. Then, we used the tunicamycin (TM) as a *N-*glycosylation inhibitor and 2-bromopalmitate (BP) as a lipidation inhibitor. We found that TM mediated the band shift of CTR1 from 30–50 kDa to 25 kDa in MM but not with BP (Fig. 6e, f, S4a-c). Further, as we expected, TM also inhibited the CTR1-plasma membrane localization (Fig. 6g, S4d) and the mutant of CTR1-*N*-glycosylation site (N23) localized to Golgi and the mutation also inhibited the band shift by glycosylation similar to TM treatment (Fig. S5a-c). These results suggested that glycosylation regulates the sub-cellular localization of CTR-1.

## Discussion

In the present study, we established a protocol for the Matrigel-based mouse MM-organoid generation as 3D culture. There are a few preceding studies on human MM, such as detection of MM stem cell markers [47, 48], recognition of transglutaminase as MM stem cell survival protein [49] and 2 human MM case studies of organoid formation by the use of photopolymerizable hyaluronic acid and gelatin hydrogen precursor to screen for appropriate chemotherapeutic drugs [50]. However, due to the rarity of human MM, it is not easy to confirm the reproducibility. Here we for the first time established the MM-organoid, using Matrigel and complete organoid medium containing basic stem cell factors (EGF, Noggin, R-spondin-1) but without FBS [51, 52]. This strategy exhibits various advantages, including EMT inhibition (Fig. S2e), preservation of CSCs and maintenance of polarity and stiffness similar to those of the actual human tumor microenvironment [12, 13]. It has been indeed established that 3D culture systems, especially CSC culture, simulate physiological conditions. Thus, our new findings could be applied to the high throughput drug screening and organoids-on-a-chip technology for targeting therapy of MM-CSC [13].

This study indeed showed an important finding that CTR1, a major cisplatin importer, localizes to the plasma membrane both in the Matrigel culture system and the implanted tumor but not in the 2D system (Figs. 5e and 6d and S2f). MβCD, a known endocytosis inhibitor, could eventually correct and induce membrane localization of CTR1. Cellular localization of CTR1 as a Cu(II) transporter dynamically changes in enterocytes where CTR1 is highly expressed [46]. Organoid culture study on CTR1 localization in intestinal cells clarified an unknown link between intestinal Cu metabolism and dietary fat processing [53]. Alterations in protein-localization are regulated at least partially by protein modifications [44, 45]. Our data also showed that plasma membrane localization of CTR1 regulates cisplatin sensitivity (Figs. 5g-h and 6e-g). Recently, it was reported that BAP1 loss in MM may modulate survival after cisplatin and/or pemetrexed treatment [54, 55]. Our RNA sequencing data showed no differences in *Bap1* gene expression between 2D culture and organoid (data not shown) but how the BAP1 plays a role in organoid culture is still unknown. Our organoid model may contribute to revealing the association between BAP1 status and cisplatin sensitivity through CTR1 localization in the near future. These findings may incite the studies not only on cisplatin-drug sensitivity through CTR1 but also on other plasma membrane proteins associated with cancer drug metabolism, including immunotherapy. In 2020, FDA approved drug combination therapy using nivolumab which can decrease tumor growth by enhancing cytotoxic T-cell function (https://www.fda.gov/news-events/press-announcements/fda-approves-drugcombination-treating-mesothelioma). For tumor cell killing, these immune cells recognize the specific ligand of immune checkpoint receptor in cancer cells [56]. These stromal cells also exist in MM tissues such as myofibroblast and macrophages (Fig. S6a). However, our organoid culture could not retain the CD68-positive macrophage population (Fig. S6b) and possibly other immune cells in the MM-organoid. A recent study showed a possibility for organoid co-culture with the immune cells to test for immunotherapy [57, 58]. For preclinical test in immunotherapy, further studies are necessary. RNA-sequence analysis revealed that the immune cells also affect to mesothelioma cells especially cytokine stimulation (Fig. S6c-d). Organoid culture, however, holds merits to quantify the ligand protein such as PD-L1, CD80, MHC and Ceacam1 because these ligand proteins are delivered to cell surface through the glycosylation, which can be demonstrated in the organoid culture. Therefore, we believe that Matrigel-based cultures would change the paradigm and improve our knowledge of cell behavior in MM.

Our RNA-sequencing data was compared in two different conditions between 2D culture in medium with FBS and antibiotics and 3D organoid under complete medium. Therefore, there is a limitation on the gene expression of our study that we cannot completely rule out the possibility that the differences might be influenced by the culture media and additives. Various culture strategies have been undertaken to establish MM cell lines [23, 24, 59]. However, MM cell lines have generally presented a problem of changing morphology into spindle-shaped via EMT [60, 61]. According with each passage, the MM cells became more spindle-shaped (data not shown). The organoid culture system could solve this issue because this system uses no FBS, thus inhibiting the EMT (Fig. S2e, f). The present protocol may be applied to the organoid culture of non-tumorous mesothelial cells as well, where the mesothelial cells can be immortalized using SV40 and small T antigen. Therefore, the formation of mesothelial organoids would be eventually possible. We first attempted to establish mesothelial organoids from mesentery (Fig. S1a, b), but the mesothelial cells without immortalization in the Matrigel failed to proliferate with essential organoid medium, resulting in a failure in passage. Further studies are in progress to focus on the ingredient requirements of the mesothelial stem-like cells and the embryonic origin of the mesothelial stem-like cells, which can be ground-breaking in this research area.

There are several reports on the MM models in mice. In this study, we used the *p53*^+/-^ mice. *CDKN2A* encodes *p16*^*INK4A*^ and *p14*^*ARF*^ (*p19*^*ARF*^ in mice), two tumor suppressors that regulate the Rb and p53 pathways, respectively. P14^ARF^ is a component of the p53 pathway and *p53* mutation has also been reported in MM [62, 63]. In this *p53*^+/-^ mouse, crocidolite injection produced MM. However, carcinogenic carbon nanotube NT-50 sometimes caused ileus due to peritonitis in addition to MM (Fig. 1b and Table 3). We used 1.5 mg NT-50 for *ip* injection, which might have been an excess dose. Previous reports on NT-50 showed that MM incidence is dose-dependent and less than 0.3 mg *ip* injection would be successful to cause MM incidence without causing peritonitis and ileus [64]. We further found that some of MM from the mouse model expressed EGFR. Three MM-cell lines from the present study were TP53-deficient and revealed EGFR expression whereas two MM-cell lines had no change in TP53 and expressed EGFR (Fig. 2d). On the other hand, our RNA-sequencing analysis showed that the tendency of *Egfr, Ctnnb1*(beta-catenin) and *Igf1r* expression was not different between organoid culture and the original tissue MM but rather intensified the tendency (Fig. S6f-h). This means that some genes are influenced on transcriptional regulation with organoid culture but others are not. EGF was also essential for MM-organoids (Fig. 2a, b) and our RNA-sequencing analysis revealed that organoid culture enriched receptor ligand activity and growth factor pathways (Fig. 4b, c). These results indicate that the organoid culture is adaptive for this tyrosine receptor kinase, including EGFR in MM.

In conclusion, we have established a novel protocol for mouse MM-organoid generation using Matrigel and the MM-organoid lines based thereon. The culture protocol would contribute to the future MM studies in mice, where EGF is required for the maintenance of MM stem-like cells in mouse MM-organoid. Finally, we believe that MM organoids can provide a pre-clinical model of humans for drug discovery and translational research in the near future.

## Supplementary Information

**Additional file 1: Figure S1.** 3D culture of cell line-derived normal mesothelial cells. **Figure S2.** m12 MM organoid reveals budding pattern. **Figure S3.** Detection of hyaluronic acid in MM-organoid. **Figure S4.** Glycosylation inhibition of CTR1 and its localization in m107 MM cells. **Figure S5.** Mutant mCTR1 protein cellular localization. **Figure S6.** Stromal cell affect cytokine activity to mesothelioma. **Figure S7.** Uncropped full-length pictures of the immunoblotting image in Figs. 2, 4 and 6. **Figure S8.** Uncropped full-length pictures of the immunoblotting image in Figure S2, S4, S5.

## Data Availability

All the relevant data supporting the results of this study can be requested to the corresponding author. The original RNA-seq data is available from the Gene Expression Omnibus (GEO) database under accession code GSE210310. A source data file has been included, which contains the raw data underlying the reported averages in all the figures and supplementary figures.

## Abbreviations

BP: 2-Bromopalmitate
CSC: Cancer stem cell
CTR1: Copper transporter 1 (SLC31A1)
EGF: Epidermal growth factor
EGFR: Epidermal growth factor receptor
EMT: Epithelial mesenchymal transition
ESC: Embryonic stem cell
FBS: Fetal bovine serum
FACS: Fluorescence-activated cell sorting
FFPE: Formalin-fixed paraffin-embedded
FGF: Fibroblast growth factor
GFP: Green fluorescent protein
GSEA: Gene set enrichment analysis
HA: Hyaluronic acid
H&E: Hematoxylin and eosin
HC: Hydrocortisone
IF: Immunofluorescence
IP: Intraperitoneal(ly)
*i*PSC: Induced pluripotent stem cell
MSLN: Mesothelin
MM: Malignant mesothelioma
PBS: Phosphate-buffered saline
PFA: Paraformaldehyde
SC: Subcutaneous(ly)
SDS-PAGE: Sodium dodecyl sulfate–polyacrylamide gel electrophoresis
TM: Tunicamycin
