# Supplementary material for: Matrigel-based organoid culture of malignant mesothelioma reproduces cisplatin sensitivity through CTR1
Source: BMC Cancer. 2023 May 31;23:487. doi: 10.1186/s12885-023-10966-4 (PMC10230733; doi:10.1186/s12885-023-10966-4)
Supplement: Supplementary file 1 — Additional file 1: Figure S1. 3D culture of cell line-derived normal mesothelial cells. Figure S2. m12 MM organoid reveals budding pattern. Figure S3. Detection of hyaluronic acid in MM-organoid. Figure S4. Glycosylation inhibition of CTR1 and its localization in m107 MM cells. Figure S5. Mutant mCTR1 protein cellular localization. Figure S6. Stromal cell affect cytokine activity to mesothelioma. Figure S7. Uncropped full-length pictures of the immunoblotting image in Figs. 2, 4 and 6. Figure S8. Uncropped full-length pictures of the immunoblotting image in Figure S2, S4, S5. [file 12885_2023_10966_MOESM1_ESM.pdf]

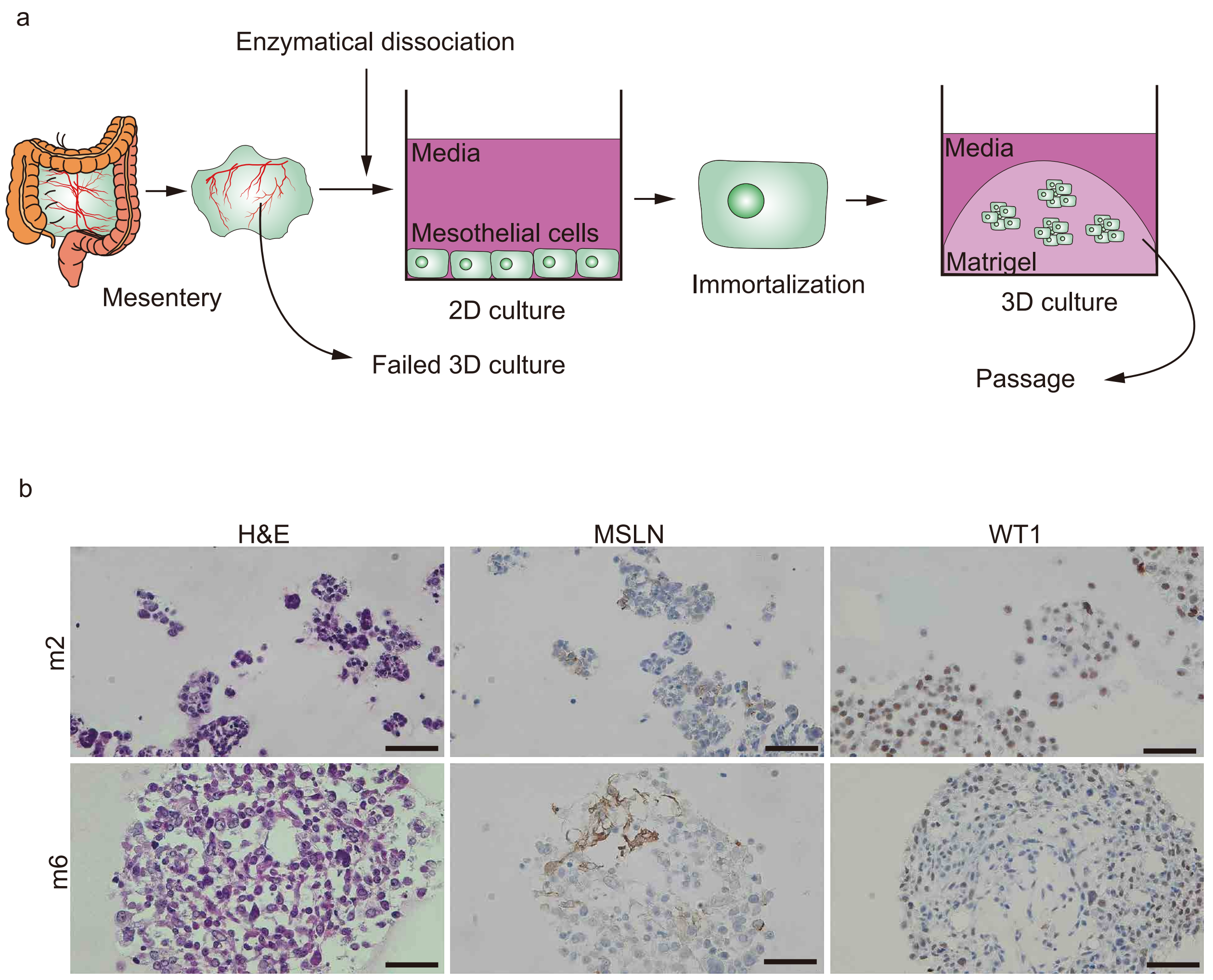
a
Enzymatical dissociation
Media
Mesothelial cells
Mesentery
2D culture
Failed 3D culture
Immortalization
Media
Matrigel
3D culture
Passage
b
H&E
MSLN
WT1
m2
m6

Figure S1

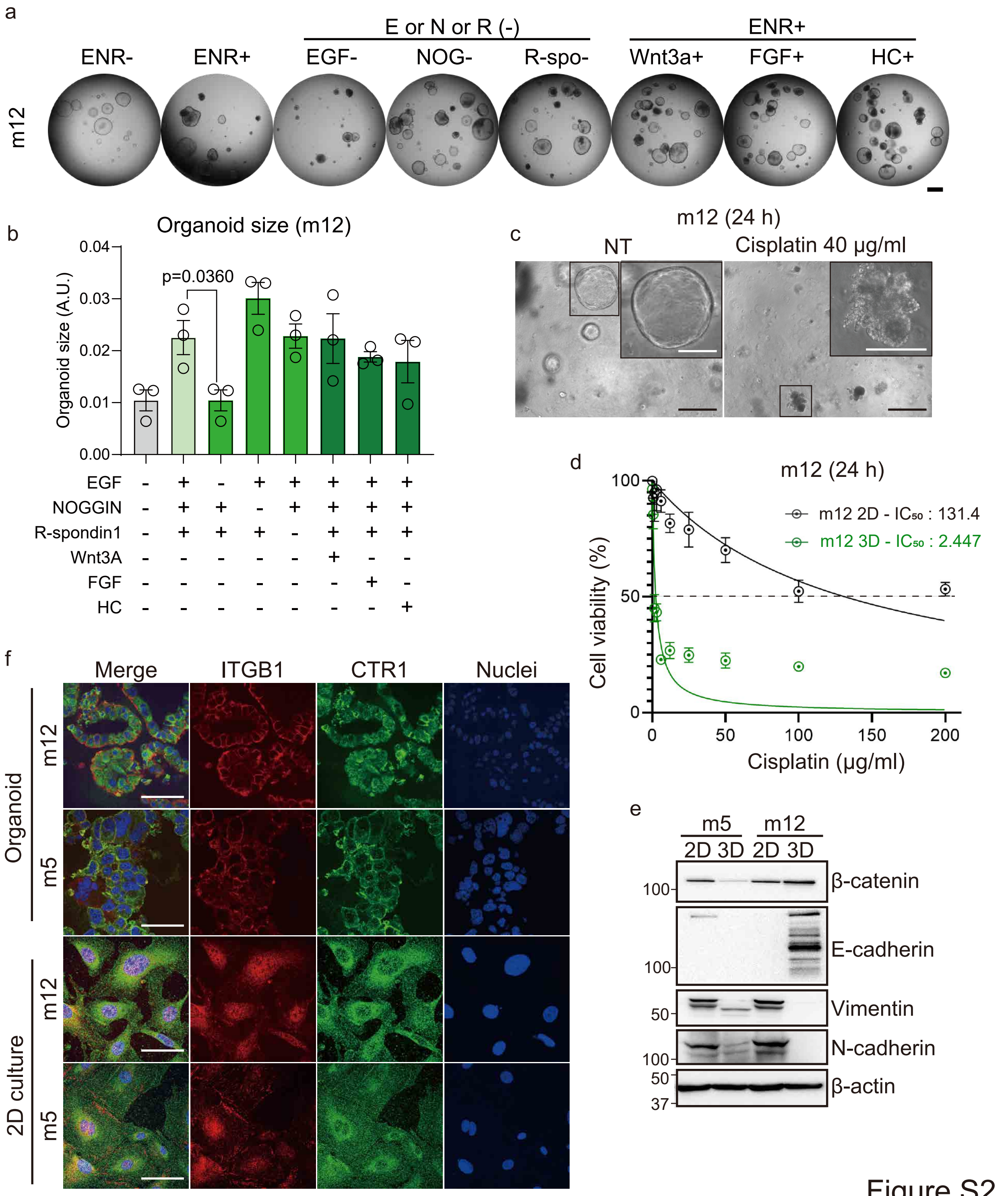
a
E or N or R (-)
ENR+
ENR-
ENR+
EGF-
NOG-
R-spo-
Wnt3a+
FGF+
HC+
m12
b
Organoid size (m12)
Organoid size (A.U.)
0.04
0.03
0.02
0.01
0.00
p=0.0360
EGF
NOGGIN
R-spondin1
Wnt3A
FGF
HC
c
m12 (24 h)
NT
Cisplatin 40 μg/ml
d
m12 (24 h)
m12 2D - IC50 : 131.4
m12 3D - IC50 : 2.447
Cell viability (%)
Cisplatin (μg/ml)
e
m5
m12
2D 3D 2D 3D
β-catenin
E-cadherin
Vimentin
N-cadherin
β-actin
f
Merge
ITGB1
CTR1
Nuclei
Organoid
2D culture
m12
m5

Figure S2

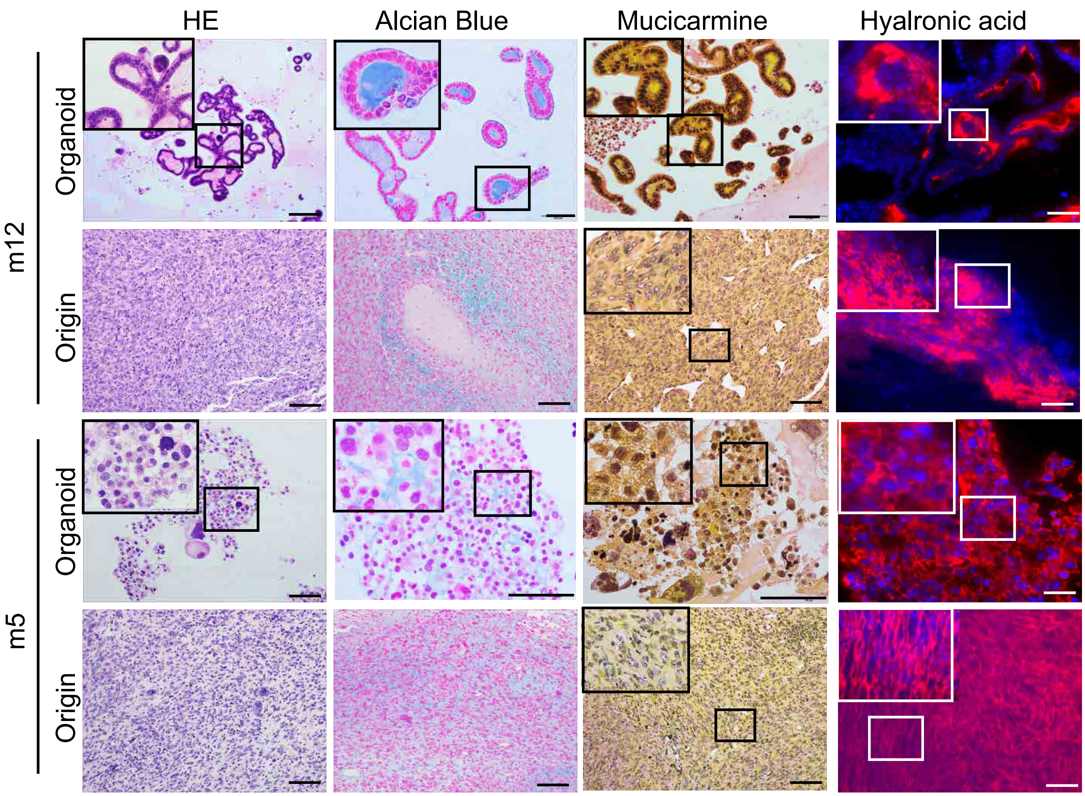
HE
Alcian Blue
Mucicarmine
Hyalronic acid
m12
Organoid
Origin
m5
Organoid
Origin

Figure S3

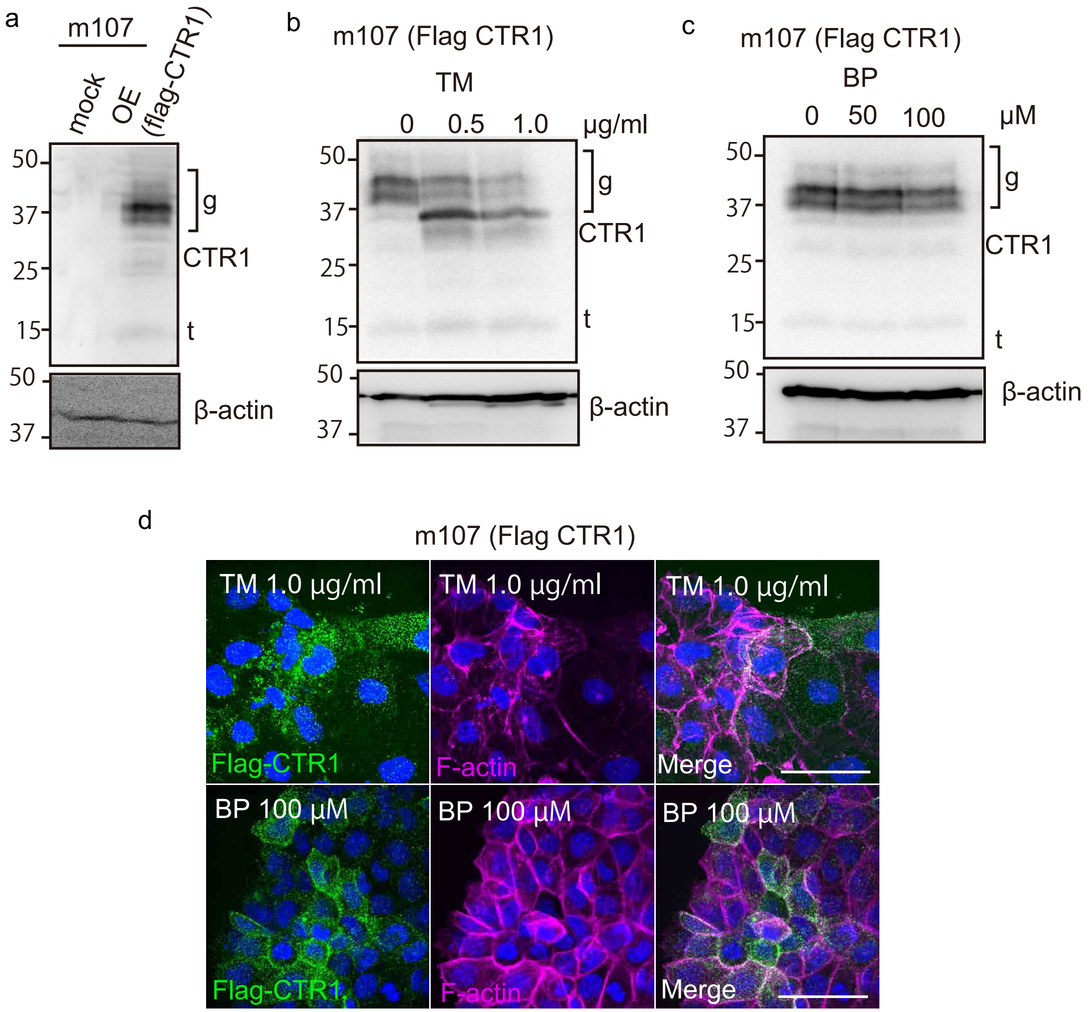
a
m107
mock
OE
(flag-CTR1)
50
37
25
15
g
CTR1
t
50
37
β-actin
b
m107 (Flag CTR1)
TM
0
0.5
1.0
μg/ml
50
37
25
15
g
CTR1
t
50
37
β-actin
c
m107 (Flag CTR1)
BP
0
50
100
μM
50
37
25
15
g
CTR1
t
50
37
β-actin
d
m107 (Flag CTR1)
TM 1.0 μg/ml
Flag-CTR1
TM 1.0 μg/ml
F-actin
TM 1.0 μg/ml
Merge
BP 100 μM
Flag-CTR1
BP 100 μM
F-actin
BP 100 μM
Merge

Figure S4

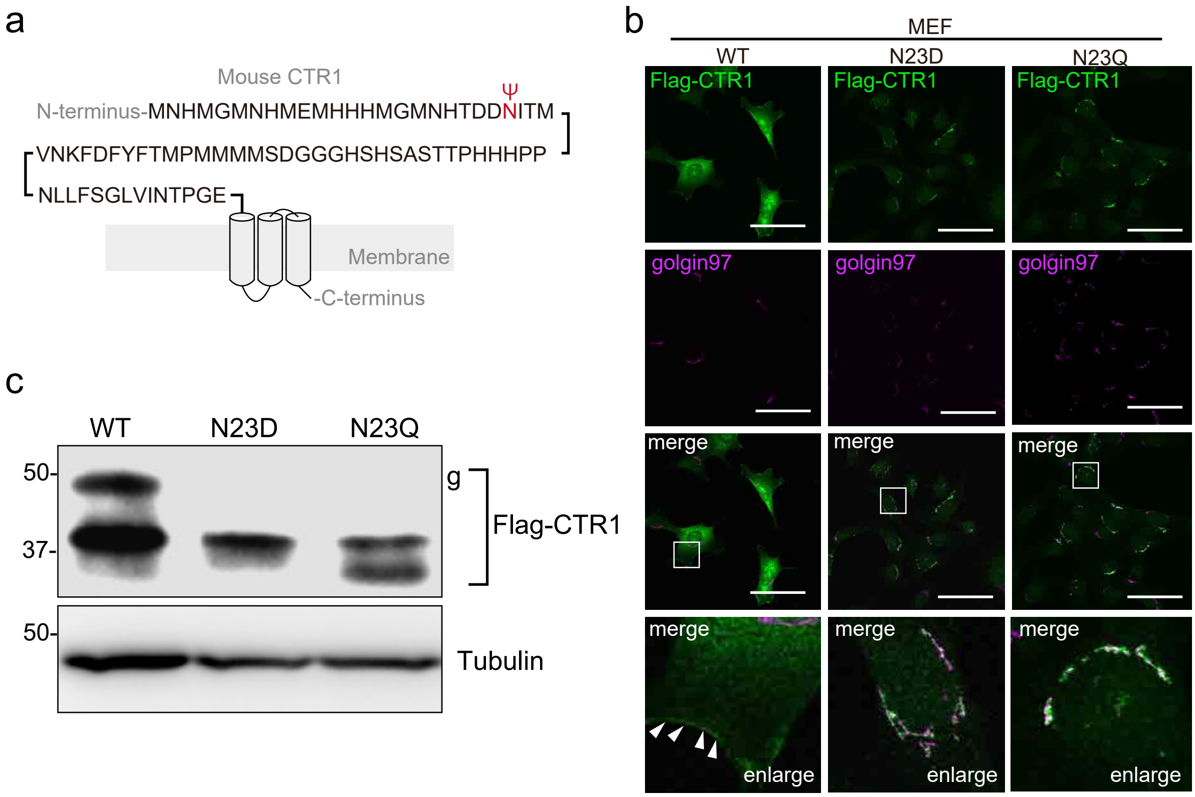
a
Mouse CTR1
N-terminus-MNHMGMNHMEMHHHMGMNHTDDNITM
VNKFDFYFTMPMMMMSDGGGHSHSASTTPHHHPP
NLLFSGLVINTPGE
Membrane
-C-terminus
b
MEF
WT
N23D
N23Q
Flag-CTR1
golgin97
merge
enlarge
c
WT
N23D
N23Q
50-
37-
g
Flag-CTR1
50-
Tubulin

Figure S5

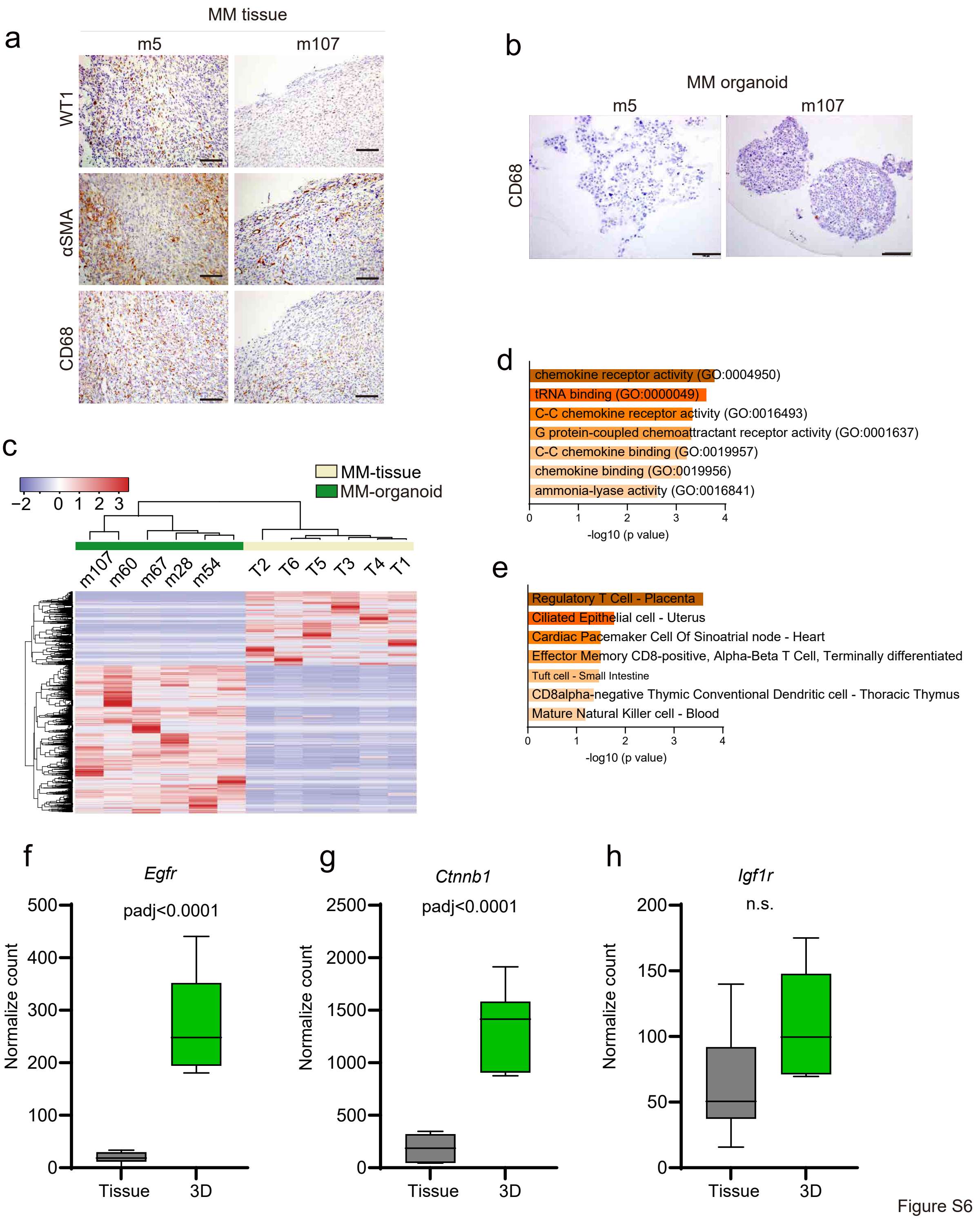
a
MM tissue
m5
m107
WT1
αSMA
CD68
b
MM organoid
m5
m107
CD68
c
MM-tissue
MM-organoid
−2 0 1 2 3
m107 m60 m67 m28 m54
T2 T6 T5 T3 T4 T1
d
chemokine receptor activity (GO:0004950)
tRNA binding (GO:0000049)
C-C chemokine receptor activity (GO:0016493)
G protein-coupled chemoattractant receptor activity (GO:0001637)
C-C chemokine binding (GO:0019957)
chemokine binding (GO:0019956)
ammonia-lyase activity (GO:0016841)
0 1 2 3 4
-log10 (p value)
e
Regulatory T Cell - Placenta
Ciliated Epithelial cell - Uterus
Cardiac Pacemaker Cell Of Sinoatrial node - Heart
Effector Memory CD8-positive, Alpha-Beta T Cell, Terminally differentiated
Tuft cell - Small Intestine
CD8alpha-negative Thymic Conventional Dendritic cell - Thoracic Thymus
Mature Natural Killer cell - Blood
0 1 2 3 4
-log10 (p value)
f
Egfr
padj<0.0001
Normalize count
0 100 200 300 400 500
Tissue 3D
g
Ctnnb1
padj<0.0001
Normalize count
0 500 1000 1500 2000 2500
Tissue 3D
h
Igf1r
n.s.
Normalize count
0 50 100 150 200
Tissue 3D

Figure S6

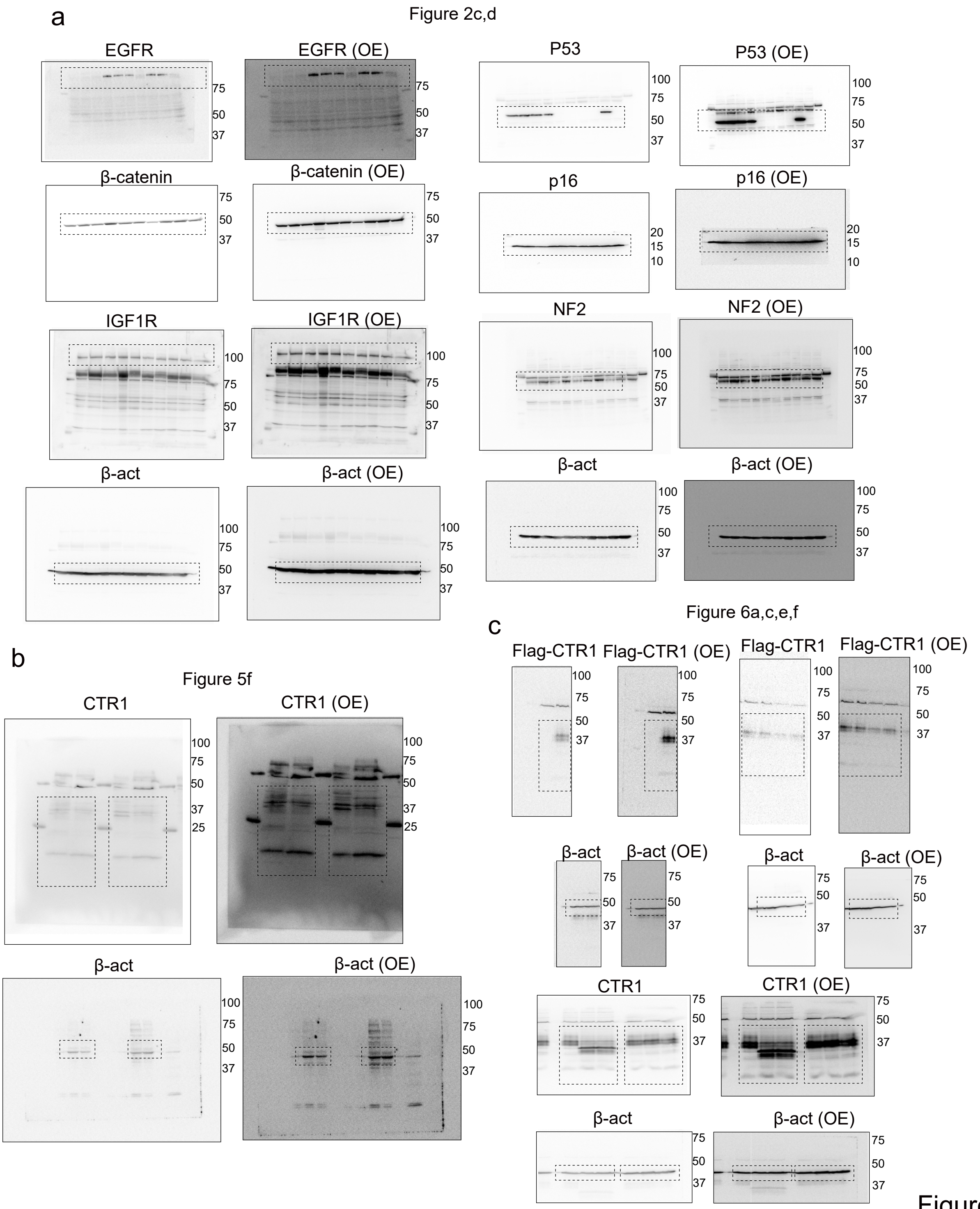
a
Figure 2c,d
EGFR
EGFR (OE)
P53
P53 (OE)
β-catenin
β-catenin (OE)
p16
p16 (OE)
IGF1R
IGF1R (OE)
NF2
NF2 (OE)
β-act
β-act (OE)
100
75
50
37
20
15
10
b
Figure 5f
CTR1
CTR1 (OE)
25
Figure 6a,c,e,f
c
Flag-CTR1
Flag-CTR1 (OE)

Figure S7

a

Figure S1e

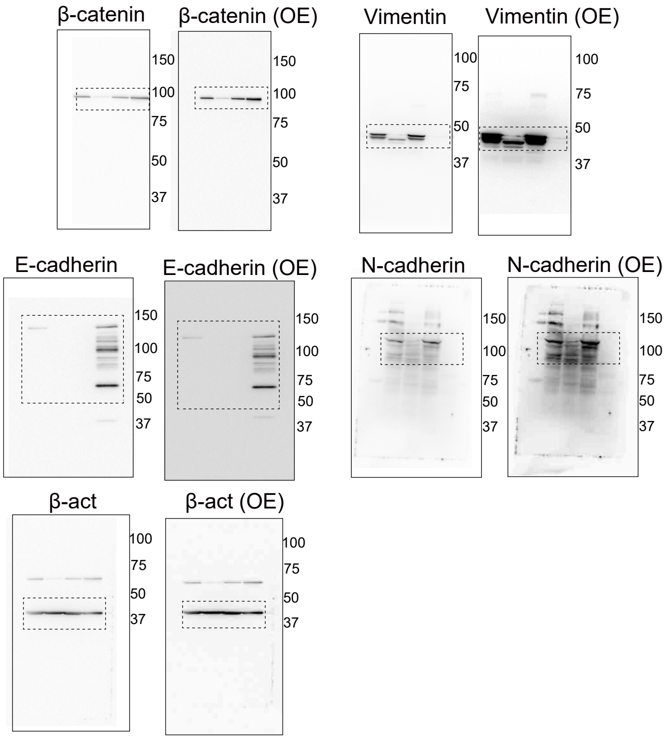

b

Figure S4a-c

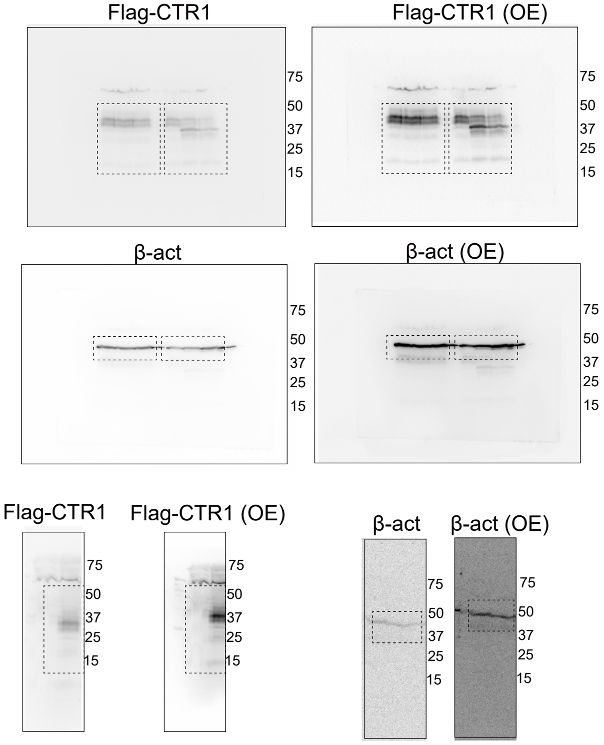

c

Figure S5c

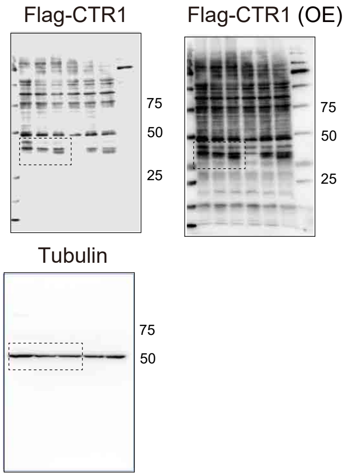

Figure S8
